# QTLs and Candidate Genes Associated with Semen Traits in Merino Sheep

**DOI:** 10.3390/ani13142286

**Published:** 2023-07-12

**Authors:** Marnie J. Hodge, Sara de las Heras-Saldana, Sally J. Rindfleish, Cyril P. Stephen, Sameer D. Pant

**Affiliations:** 1 School of Agricultural, Environmental and Veterinary Sciences, Charles Sturt University, Wagga Wagga, NSW 2678, Australia; mhodge@csu.edu.au (M.J.H.); cstephen@csu.edu.au (C.P.S.); 2 Apiam Animal Health, Apiam Genetic Services, Dubbo, NSW 2830, Australia; jane.rindfleish@gmail.com; 3 Animal Genetics and Breeding Unit, a Joint Venture of NSW Department of Primary Industries and University of New England, Armidale, NSW 2351, Australia; sdelash2@une.edu.au; 4 Gulbali Institute, Charles Sturt University, Boorooma Street, Wagga Wagga, NSW 2678, Australia

**Keywords:** ejaculate quality, ovine, GWAS, ram, reproduction

## Abstract

**Simple Summary:**

Ram semen traits including volume, gross motility, concentration, and percent post-thaw motility are routinely assessed prior to use in artificial breeding programs, as they have been shown to influence conception outcomes. Semen quality and associated traits are complex but heritable, and as such, identifying genes that underlie variability in these traits may help develop alternative means to improve conception outcomes and therefore reproductive efficiency in sheep. Therefore, the aim of this study was to identify genomic regions and associated genes that may significantly influence semen traits like volume, gross motility, concentration, and percent post-thaw motility in Merino sheep. Assessment of over 20 years’ worth of semen collection data identified 35 genomic regions to be significantly associated with Merino ram semen volume, gross motility, concentration, and percent post-thaw motility. A total of 290 candidate genes were identified within genomic regions found to be significantly associated with Merino ram semen traits. All Merino ram semen traits were also found to be lowly heritable, affirming results from previous studies. Validation of candidate genes identified in the current study could provide novel insights into molecular mechanisms contributing to variability in semen associated traits.

**Abstract:**

Ram semen traits play a significant role in conception outcomes, which in turn may influence reproductive efficiency and the overall productivity and profitability of sheep enterprises. Since hundreds of ewes may be inseminated from a single ejaculate, it is important to evaluate semen quality prior to use in sheep breeding programs. Given that semen traits have been found to be heritable, genetic variation likely contributes to the variability observed in these traits. Identifying such genetic variants could provide novel insights into the molecular mechanisms underlying variability in semen traits. Therefore, this study aimed to identify quantitative trait loci (QTLs) associated with semen traits in Merino sheep. A genome-wide association study (GWAS) was undertaken using 4506 semen collection records from 246 Merino rams collected between January 2002 and May 2021. The R package RepeatABEL was used to perform a GWAS for semen volume, gross motility, concentration, and percent post-thaw motility. A total of 35 QTLs, located on 16 *Ovis aries* autosomes (OARs), were significantly associated with either of the four semen traits in this study. A total of 89, 95, 33, and 73 candidate genes were identified, via modified Bonferroni, within the QTLs significantly associated with volume, gross motility, concentration, and percent post-thaw motility, respectively. Among the candidate genes identified, *SORD*, *SH2B1*, and *NT5E* have been previously described to significantly influence spermatogenesis, spermatozoal motility, and high percent post-thaw motility, respectively. Several candidate genes identified could potentially influence ram semen traits based on existing evidence in the literature. As such, validation of these putative candidates may offer the potential to develop future strategies to improve sheep reproductive efficiency. Furthermore, Merino ram semen traits are lowly heritable (0.071–0.139), and thus may be improved by selective breeding.

## 1. Introduction

Reproductive efficiency in livestock depends on multiple factors, including insemination, conception, embryonic development, and parturition, and is a key determinant of farm productivity and profitability [1,2]. Reproductive efficiency is influenced by an interplay between the environment and host genetics, which in turn includes both male- and female-side genetic determinants. In this context, significant emphasis has been placed on identifying genetic variants associated with female reproductive efficiency in livestock species in the past [3,4,5], while relatively little emphasis has been placed on identifying male-side genetic variants influencing livestock reproductive efficiency. Furthermore, there is only one past sheep study that has investigated the influence of genetic variants associated with semen traits, which was performed using a sheep breed that is not common across Australia [6]. In livestock species such as sheep, a single ram has the potential to produce hundreds of progeny annually via natural breeding or artificial insemination (AI) [7]. For example, top-ranking Merino sires on different selection indices in MERINOSELECT frequently have hundreds of progeny recorded across multiple flocks [8]. Therefore, it is important to assess the breeding soundness of rams prior to natural breeding, or alternatively, assess semen quality prior to artificial breeding programs. Likewise, the sire has a significant influence on the number of lambs born, with the breed of ram also influencing prenatal mortality [9]. Poor conception outcomes, in turn, may result in fewer lambs being born and may also potentially extend the joining and lambing periods, which could have subsequent negative impacts on the productivity and profitability of sheep farming enterprises.

Ram semen traits are significantly influenced by a variety of factors, including time of year [10,11], nutrition [12], semen collection method [13], and seminal plasma [14,15,16]. Studies have also demonstrated that ovine spermatozoa contain a variety of transcripts [17], but the relevance of these in the context of semen traits has not yet been determined. Specifically in sheep, semen traits such as gross motility [18], concentration [19], and percent post-thaw motility [20] appear to influence conception outcomes, which in turn could significantly influence the profitability of sheep farming enterprises. However, low heritabilities have been reported for ram semen volume (0.08–0.20 [21], 0.161 [22], 0.18–0.33 [23], 0.07–0.11 [24]), gross motility (0.03–0.11 [21], 0.170 [22], 0.07–0.14 [23], 0.32–0.27 [24]), concentration (0.10–0.19 [21], 0.089 [22], 0.18–0.27 [23], N/A–0.17 [24]), and percent post-thaw motility (0.081 [22]). This indicates that a diverse range of factors, including host genetics, contribute to the variability in these traits. Identifying genetic variants that contribute to variability in these traits could assist in characterising the underlying molecular mechanisms, which in turn could help to develop alternative strategies to improve reproductive efficiency in sheep farming enterprises. 

In the context of semen traits, previous genome-wide association studies (GWAS) have identified several QTLs and candidate genes in a range of livestock species, including dairy sheep [6], goats [25], cattle [26,27,28,29,30,31], and pigs [32,33,34,35,36,37]. For example, a GWAS in Assaf sheep reported 20, 23, 76, and 32 single nucleotide polymorphisms (SNPs) on chromosomes 3, 17, 4, and 1 to be associated with semen volume, gross motility, concentration, and number of spermatozoa per ejaculate, respectively [6]. In the same study, *SLC9C1* and *FUT10* were identified as putative candidate genes for gross motility due to their location within identified QTL regions, and because of previous reports that these genes influenced spermatozoal motility, hyperactivation, and fertilisation, respectively. Similarly, a GWAS involving semen volume and number of sperm collected from Holstein-Friesian bulls identified several candidate genes such as *GALC*, *PHF7*, and *PRKCD*, all of which have been previously identified to play a role in spermatogenesis [38]. Similar investigations in pigs have identified several putative candidates associated with post-thaw motility and membrane integrity, including *PLBD1*, *OXSR1*, and *EML5* [39], and polymorphisms in such genes have been previously reported to be associated with fertilisation in humans [40], spermatozoal motility in pigs [41], and spermatogenesis in bulls [42], respectively. Despite several past GWAS focused on identifying genomic regions and candidate genes associated with semen traits across a wide range of livestock species, the only previous study in sheep was conducted using Assaf rams [6], and there are no studies involving other common sheep breeds like the Merino. While it is likely that the physiology associated with semen traits is mechanistically conserved across species, the genes that contribute to variability in these traits may vary depending on the genetic background of any given population of animals. In Australia, Merino sheep account for 74% of Australia’s ewe breeding flock and 56% of the lamb flock, with 42.4 million Merino ewes bred annually [43]. Therefore, the objective of this study was to perform a genome-wide association study to identify QTL regions and associated candidate genes that significantly influence semen traits in Merino rams. 

## 2. Materials and Methods

### 2.1. Phenotype Data

Phenotype data, including semen volume, gross motility, concentration, and percent post-thaw motility, was obtained from a single commercial artificial breeding company (which had two centres over time). The collection and assessment of semen quality, descriptive statistics, and data quality control have been previously described [22]. The dataset for Genome-Wide Association (GWA) analysis consisted of a total of 4506 semen collection records from 246 Merino rams that had been previously genotyped by their flock owners (hence, 246 rams had both semen phenotype data and had been genotyped). Descriptive statistics for semen traits (Table 1) also fall within the normal ranges of semen traits for ram [7]. 

### 2.2. Genotype Data and Quality Control

Genotype data for 246 rams included in this study was provided by Sheep Genetics (Meat & Livestock Australia). Animals were genotyped using different Ovine SNP chips ranging from 12k (n = 31), 15k (n = 81), 50k (n = 122), and 700k (n = 12). Low-density chips were subsequently imputed to 50k using a reference population as part of the routine pipeline followed by the Animal Genetics and Breeding Unit (AGBU), which was previously described [44]. Briefly, Beagle software v3.2 [45] was used to impute 12k and 15k to 50k SNPs. PLINK [46] was then used to perform quality control, excluding SNPs based on the following criteria: < 0.01, Hardy–Weinberg equilibrium *p*-value < 1 × 10^−6^, and if they were located on sex chromosomes. In total, 58,946 SNPs were used for subsequent GWA analysis. The genomic positions of SNPs were taken from the ovine (*Ovis aries*) reference genome build (Oar_v3.1).

### 2.3. Model for Analysis

#### 2.3.1. Statistical Model

Preliminary analysis was performed to assess the significance of each fixed effect for individual semen traits via linear regression using the R software package, version 4.1.2 [47]. The fixed effects that were assessed included age of collection (n = 8), collection centre (n = 2), season (n = 4), method of collection (n = 2), and collection number. Once the fixed effects that significantly influenced each semen trait were identified (Supplementary Table S1), a linear mixed model was used to perform genome-wide association analysis using a model that included several fixed effects, a random polygenic effect, and the random effect associated with the stud of breeding and year of collection:

$$y=u+X_{SNP}\beta_{SNP}+Z_{g}+Z_{p}+e$$

where y represents the vector of transformed phenotypes for individual semen traits; u is an intercept term; $X_{SNP}$ and $\beta_{SNP}$ represent SNP dosage and effects, respectively; $Z_{g}$ and $Z_{p}$ are incidence matrices relating to the individuals and their observed values for random polygenic and random stud-year effects; and e represents the residuals [48].

#### 2.3.2. Identification of SNPs and Associated Candidate Genes

The R package RepeatABEL v1.1 [48] was used for GWA analysis, as it allows for the analysis of datasets with repeated measures. A ‘prefitModel’ function was initially used in RepeatABEL by fitting a linear mixed effect model that included the genomic relationship matrix (GRM). This accounted for random polygenic effects without including SNP effects. Secondly, the ‘rGLS’ function was used in RepeatABEL to test for SNP association by accounting for the fixed (Supplementary Table S1) and random effects of fitted models for each semen trait. The *p*-values for each SNP, as well as the SNP effect of each marker, were determined via Wald statistics within the RepeatABEL software package. Finally, to accurately account for multiple testing levels, the significance threshold was calculated via modified Bonferroni [49] as follows:

$$\frac{\alpha }{\sqrt{nSNP}}$$

where α = 0.05 and nSNP is the total number of SNPs identified across each of the four semen traits.

Candidate genes were identified in QTL regions that spanned 0.5 Megabase (Mb) around each significant SNP (*p*-value < 0.0002). These QTL regions were used to search for candidate genes through the National Centre of Biotechnology Information (NCBI) Genome Data Viewer [50] using the ovine (*Ovis aries*) genome assembly (Oar_v3.1). 

The SNP variance was calculated as the total variance (as previously described [51]) explained by each SNP as a proportion of the additive genetic variance using the following formula:

$$V_{SNP}\%=100\times \frac{2pqa^{2}}{\sigma_{a}^{2}}$$

where p and q are the major and minor allele frequencies, respectively; a is the additive SNP effect; and $\sigma_{a}^{2}$ is the additive genetic variance.

### 2.4. Heritability and Variance Component Estimation of Semen Phenotypes

Heritability and variance components were estimated for semen volume, gross motility, concentration, and percent post-thaw motility using the ‘prefitModel’ in the ‘rGLS’ function, including the GRM, via the R package RepeatABEL [48]. The same significant fixed effects used for the GWAS for each semen phenotype were fitted in the model, as defined (Supplementary Table S1). Heritability (h^2^) of semen phenotypes was calculated as follows:

$$h^{2}=\frac{\sigma_{a^{2}}}{\sigma_{a^{2}}+\sigma_{c^{2}}+\sigma_{d^{2}}+\sigma_{e^{2}}}=\frac{\sigma_{a^{2}}}{\sigma_{{pe}^{2}}}$$

where $\sigma_{a^{2}}$ is the estimate for additive genetic variance; $\sigma_{c^{2}}$ is the variance estimate for random permanent environmental genetic effects; $\sigma_{d^{2}}$ is the estimate of random stud-year effect; $\sigma_{e^{2}}$ is the estimate of the residual error variance; and $\sigma_{{pe}^{2}}$ is the phenotypic variance (being the sum of additive genetic, random permanent environmental, stud-year, and residual variance effects). 

Estimates of repeatability (r) were calculated as follows:

$$r=\frac{\sigma_{a^{2}}+\sigma_{c^{2}}+\sigma_{d^{2}}}{\sigma_{{pe}^{2}}}$$

where $\sigma_{a^{2}}$, $\sigma_{c^{2}}$, $\sigma_{d^{2}}$, and $\sigma_{{pe}^{2}}$ have been previously defined.

## 3. Results

**Figure 1:**
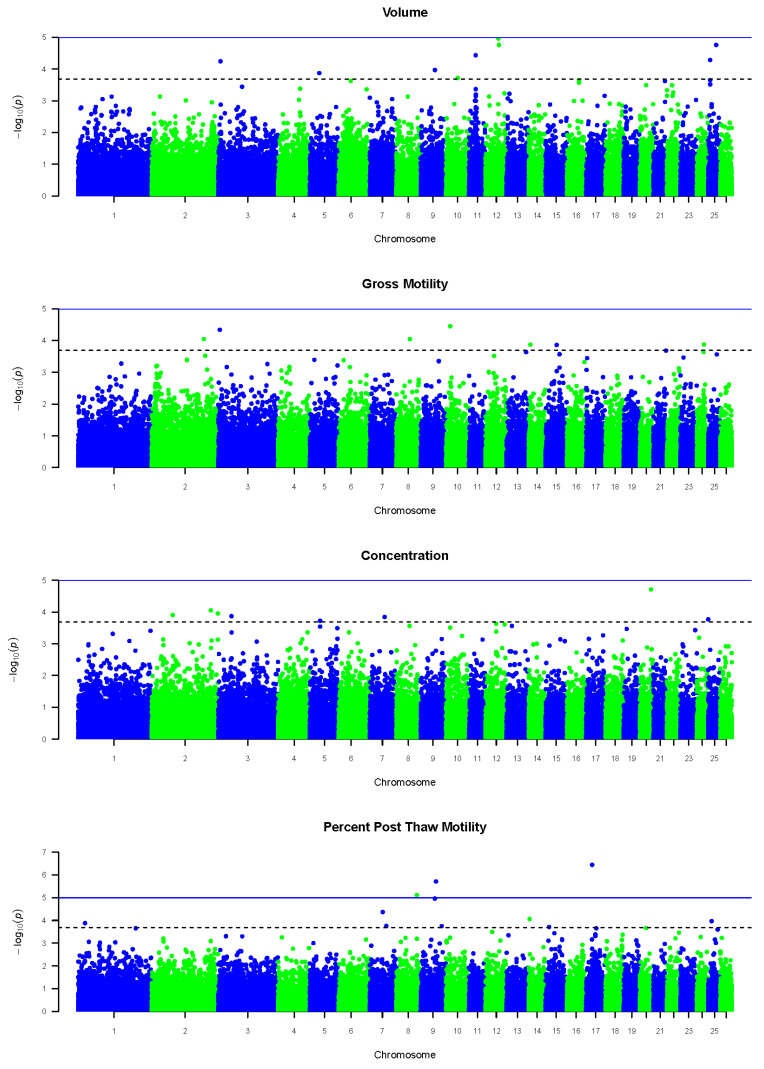
Manhattan plots depicting the distribution of total SNPs significantly associated with volume, gross motility, concentration, and percent post-thaw motility across *Ovis aries* autosomes (OARs), with the solid blue line representing the genome-wide significance threshold and the dotted grey line representing the modified Bonferroni threshold.

Several SNPs were found to be significantly associated with each semen trait included in this study, i.e., volume, gross motility, concentration, and percent post-thaw motility. Manhattan plots associated with GWA analysis of each of the semen traits are presented in Figure 1.

A total of 35 significant SNPs were found across a total of 16 OARs (Table 2). Of those, nine, seven, eight, and eleven SNPs were associated with volume, gross motility, concentration, and percent post-thaw motility, respectively. Furthermore, SNPs for semen volume, gross motility, concentration, and percent post-thaw motility were found across seven, six, six, and eight unique OARs, respectively. The SNPs with the highest significance identified in each semen trait were 12:48,412,883, 10:16,620,353, 20:42,505,407, and 17:22,669,197 for semen volume, gross motility, concentration, and percent post-thaw motility, respectively. 

Overall, 143, 141, 97, and 138 candidate genes were identified within significant genomic regions associated with semen volume, gross motility, concentration, and percent post-thaw motility after searching the QTL regions, which spanned 0.5 Megabase (Mb) around each significant SNP associated with semen traits via the NCBI Genome Data Viewer using the Oar_v3.1 genome assembly. Several genes located within QTLs were not annotated or did not have known orthologs, and as such, these genes had a ‘LOC’ prefix. A summary of all these genes is presented in Supplementary Table S2. Therefore, a total of 35 QTLs, specifically nine, seven, eight, and eleven regions, were associated with volume, gross motility, concentration, and percent post-thaw motility, respectively.

Table 3 presents candidate genes identified within each QTL region associated with semen volume, gross motility, concentration, and percent post-thaw motility. Unique candidate genes found within QTL regions for each of the semen traits include 89 for volume, 95 for gross motility, 33 for concentration, and 73 for percent post-thaw motility. Several candidate genes were identified within the most significant QTLs for each semen trait, including volume *PRDM16*, *ACTRT2*, *TRNAC-GCA*, *TTC34*, *MMEL1*, *FAM213B*, *HES5*, *PANK4*, *PLCH2*, *PEX10*, *RER1*, *MORN1*, *SKI*, *C12H1orf86*, *PRKCZ*, *GABRD*, *CFAP74*, *TMEM52*, *CALML6*, gross motility; *SPERT*, *SIAH3*, *ZC3H13*, *CPB2*, *LCP1*, *LRRC63*, *KIAA0226L*, *LRCH1*, *ESD*, *TRNAG-CCC*, and concentration; *SIRT5*, *GFOD1*, *TBC1D7*, *PHACTR1*, and percent post-thaw motility; no candidate genes identified within QTL.

The heritability estimates for semen volume, gross motility, concentration, and percent post-thaw motility are presented in Table 4. Heritability estimates for volume and gross motility are lowly moderate (0.104 and 0.139, respectively), while concentration and percent post-thaw motility have low heritability estimates (0.071 and 0.092, respectively). The additive genetic, environmental, residual, and random effects variances are higher for concentration in comparison to the other semen traits.

## 4. Discussion

In sheep, semen traits including concentration [19], gross motility [18], and percent post-thaw motility [20] are reported to influence conception outcomes, but the underlying mechanisms are not well understood. Identifying genes and genomic regions associated with such traits can help provide novel insights into these mechanisms. Therefore, the aim of this study was to investigate regions on the ovine genome and candidate genes associated with Merino ram semen traits including volume, gross motility, concentration, and percent post-thaw motility.

This study identified a total of 35 QTLs located on 16 OARs, specifically nine, seven, eight, and eleven QTLs for semen volume, gross motility, concentration, and percent post-thaw motility, respectively (Table 3). The nine QTLs identified to be associated with semen volume were located on OARs three, five, nine, ten, eleven, twelve, and twenty-five. Eighty-nine unique candidate genes were identified within these QTLs for semen volume. Of these, several candidates were noteworthy because they have previously been reported to be expressed within testes and sperm cells as well as associated with spermatozoal maturation. Examples of such genes include *solute carrier family 2 member 8* (*SLC2A8*), *actin-related protein T2* (*ACTRT2*), *ribosomal protein L12* (*RPL12*), and *MIS12 kinetochore complex component* (*MIS12*). *SLC2A8*, also known as *Glucose transporter 8* (*GLUT8*), is highly expressed in the testes and is located within the acrosome of spermatozoal cells as well as in the plasma membrane [52]. In the same study, *SLC2A8* mRNA synthesis was found to coincide with spermatozoal maturation in the mouse testes. In other murine studies, spermatozoal motility was significantly impaired in *SLC2A8* null mice due to reduced mitochondrial function [53], while oocytes harvested from *SLC2A8* null mice had irregular changes to the endometrium during embryo implantation [54]. As such, *SLC2A8* may be a putative candidate for ram spermatogenesis and motility. Another such gene, *ACTRT2*, is reported to be localised in the post-acrosomal region of mouse spermatozoa [55], as well as the post-acrosomal region and midpiece in males with low spermatozoal motility which resulted from obesity [56]. Therefore, *ACTRT2* may play a key role in the normal morphological development of the acrosome and membrane integrity, as well as promoting spermatozoal motility. Proteomic analysis of ram semen identified ACTRT2 as being significantly expressed in fresh semen compared to cryopreserved semen [57]. In a pig study, *ACTRT2* was significantly downregulated in fresh semen collected during the non-breeding season [58], which is associated with reduced semen quality in pigs [59]. As time of year influences semen quality in both pigs [60,61] and sheep [10,11], *ACTRT2* may be a key gene modulating semen quality during the breeding and non-breeding seasons. Another gene, *RPL12*, is known to be significantly downregulated in bulls with low fertility, as assessed via conception rate following artificial insemination [62]. *RPL12* has also been found to be upregulated in ram testes [63]. Given that spermatogenesis, maturation of spermatozoa, and spermatozoal motility occur within the testes [64], *RPL12* may be a positional candidate gene that plays a key role in promoting ram semen maturation and motility. MIS12 has been previously reported to be enriched within networks associated with chromatin condensation following proteomic analysis of turkey testes [65]. Given that chromatin condensation is correlated with the fertilising ability of a sperm [66,67], *MIS12* may play a crucial role in sperm quality and conception success. Furthermore, proteomic analysis of seminal plasma identified MIS12 as being commonly expressed across all fertile males [68]. As semen consists of spermatozoa and seminal plasma, which significantly enhance ram sperm motility and membrane integrity [69], *MIS12* may be a putative candidate gene within ram seminal plasma that may modulate sperm motility and viability. Thus, associated positional candidate genes identified in QTLs significant for semen volume may also have functional roles in overall semen quality.

Seven QLTs, located on six different OARs, were significantly associated with gross motility. A total of 95 candidate genes were identified within these seven QTL regions, of which several key genes have been previously associated with spermatogenesis and semen quality, including *SH2B adaptor protein 1* (*SH2B1*), *mitogen-activated protein kinase 3* (*MAPK3*), *aldolase*, *fructose-bisphosphate A* (*ALDOA*), *5′-nucleotidase ecto* (*NT5E*), and *spermatid associated protein* (*SPERT*). *SH2B1* may be a putative candidate influencing spermatozoal motility since *SH2B1* was identified within one of the significant QTLs (18.51–18.87 Mb on *Sus scrofa* chromosome 3) associated with progressive motility in Duroc boars [33]. Furthermore, reduced mRNA expression of *SH2B1* in obese mice resulted in reduced motility and hyperactivation compared to control mice [70]. Another gene, *MAPK3*, is associated with spermatozoal hyperactivation [71], which is a crucial process required prior to sperm fertilising an ova [72]. Moreover, *MAPK3* was also identified as a common differentially expressed gene (DEG) in semen collected from Merino rams when contrasting breed specific differences in the spermatozoal transcriptome [17], as well as a candidate gene associated with sire fertility traits following a GWAS in Holstein cattle [73]. Hence, *MAPK3* may be a key putative candidate underlying ram spermatozoal motility. Another candidate, *ALDOA*, was abundantly expressed in spermatozoa collected from rams with high motility compared to rams with low motility [74]. Likewise, *ALDOA* was abundantly expressed in rams with higher pregnancy rates following natural mating compared to rams with lower pregnancy rates across three breeding seasons [75]. This suggests that expression of *ALDOA* may contribute to spermatozoa motility in rams and thus may be a key putative candidate for spermatozoal motility. Proteomic analysis of ram spermatozoa identified NT5E to be abundantly expressed in ram semen with high motility following cryopreservation (and therefore, a high tolerance to cryopreservation) compared to semen with a low cryo-tolerance [76]. Therefore, *NT5E* may be a key functional candidate for spermatozoal motility and cryo-tolerance in sheep. Additionally, *NT5E* was found to be enriched in both seminal plasma and seminal vesicle fluid of bulls [77]. Finally, *SPERT*, also known as *chibby family member 2* (*CBY2*), was significantly expressed in the testes collected from buffalo during puberty compared to testicular tissue from both pre- and post-pubertal buffalo [78]. In the same study, *SPERT* was found to be localised to the acrosome, neck, and midpiece of buffalo spermatozoa. As such, *SPERT* may be a key candidate for spermatogenesis and spermatozoal morphology, given that it is significantly expressed during puberty and is localised to the sperm head and midpiece.

Thirty-three candidate genes were identified within eight QTLs, located on six OARs, that were found to be associated with semen concentration. Some candidate genes identified in this study have previously been reported in studies related to spermatogenesis and testicular development, including *peptidyl arginine deiminase 2* (*PADI2*), *cytochrome P450*, *family 19* (*CYP19*), and *actin like 8* (*ACTL8*). *PADI2* may be a putative candidate gene influencing spermatozoal concentration and motility since *PADI2* is abundantly expressed in the epididymis, predominantly in the caput epididymis and vas deferens following RNA sequencing of murine testes [79]. In several mammalian species, transit through the epididymis is crucial for spermatozoal maturation and attainment of motility [80,81,82,83], as well as ensuring adequate concentration of spermatozoa in semen through fluid absorption [84]. Hence, *PADI2* may be a putative candidate associated with spermatozoal concentration and a positional candidate modulating spermatozoal maturation and motility. In the same context, *PADI2* was found within one of ten *Bos taurus* autosomes (BTA), explaining the highest portion of genetic variance for both total and progressive motility (BTA 2:1349.81–1359.81) [29]. In the current study, *PADI2* is located within OAR 2:247.37–248.37, and as such, *PADI2* may be a significant putative candidate for spermatozoal motility in Merino sheep. Furthermore, *PADI2* was identified within a QTL associated with the number of piglets born dead and alive [85]; hence, *PADI2* may play a key role in litter size and post-parturition offspring survival in livestock. Another noteworthy candidate gene, *CYP19*, is associated with spermatozoal concentration and motility in humans, with polymorphisms of *CYP19* resulting in reduced sperm concentration within normozoospermic males [86]. The authors suggested that altered aromatase concentrations within the testes resulted in reduced concentrations of spermatozoa within normozoospermic males. Aromatase promotes oestrogen production in humans and mice [87,88,89] and is found to be expressed in several cell types, e.g., Sertoli and Leydig cells, spermatids in rats [90], as well as semen in humans [91,92]. A past pig study revealed *CYP19* to be abundantly enriched in spermatozoa, which had higher embryonic cleavage rates compared to spermatozoa, which had lower cleavage rates [93]. Therefore, *CYP19* may be a key candidate for modulating the concentration of spermatozoa in an ejaculate. Expression of *ACTL8* has been reported to be isolated to the testes following a study that undertook exome sequencing in males with non-obstructive azoospermia [94], a condition where no sperm is present in an ejaculate due to failures during spermatogenesis [95]. In the same study, mutations in *ACTL8* were not present in the control patients. Hence, *ACTL8* may significantly influence spermatogenesis and spermatozoal concentration within an ejaculate. Thus, noteworthy genes identified in QTLs significantly associated with concentration of Merino ram semen, such as *PADI2*, *CYP19*, and *ACTL8*, may be putative candidates for semen traits like concentration and motility in sheep.

A total of eleven QTLs on eight different OARs were found to be significantly associated with percent post-thaw motility. A total of 73 candidate genes were identified within these QTL regions. Of these 73 candidates, several have previously been reported to be associated with semen quality and male-side influence on conception outcomes, including *MON1 homolog B*, *secretory trafficking associated* (*MON1B*), *RAB3B*, *member of the RAS oncogene family* (*RAB3B*), *ribosomal protein L30* (*RPL30*), and *sorbitol dehydrogenase* (*SORD*). *MON1B*, located on OAR 14, was identified within genomic regions associated with both cow- and daughter-conception rates following a GWAS in Holstein bulls with known conception rates [96]. In a similar study, *MON1B* was found to be one of twelve genes associated with embryonic development following a bovine GWAS for cryotolerance and embryonic development in cattle [97]. Thus, *MON1B* may be an important candidate gene that may modulate conception success. RNA Sequencing identified *RAB3B* as being significantly expressed in good-quality semen compared to low-quality semen following assessment of semen traits in bulls [98]. Hence, *RAB3B* may be a key putative candidate for modulating spermatozoal motility in sheep. Moreover, RAB3-peptides, including *RAB3B*, are key regulators of the acrosome reaction in sheep [99]. Given that the acrosome reaction is a necessary process for fertilisation [100], *RAB3B* may be an important candidate gene for promoting sperm binding to the ova and promoting conception success. In a bull study, *RPL30* was abundantly expressed in bulls with high sperm motility compared to bulls with low motility [101]. Moreover, *RPL30* was up- and downregulated in normozoospermic and asthenozoospermic males, respectively, compared to controls [102]. Hence, *RPL30* may be a key putative candidate gene modulating spermatozoal motility. Another gene, *SORD*, was identified within the flagella of sperm extracted from the cauda epididymis and had increased expression during the late stages of spermatogenesis in mice [103]. In the same study, spermatozoa collected from the cauda epididymis incubated in sorbitol exhibited motility after 4 h of incubation, compared to control spermatozoa. In a sheep study, SORD was identified as a key candidate in cryotolerance following proteomic analysis of ram seminal plasma [15]. Hence, *SORD* may play a role in modulating ram spermatozoal motility and cryotolerance and, therefore, may influence post-thaw motility in sheep.

While there are several published GWAS on semen traits in livestock, only a single study has been published in sheep, which involves identifying QTL regions associated with semen volume, concentration, gross motility, and number of spermatozoa using semen records collected from 429 Assaf rams [6]. There is no overlap between QTLs identified in the present study and QTL regions associated with Assaf ram semen volume, concentration, and gross motility [6]. While the present study identified 35 QTLs, this was based on a relatively lenient genome-wide significance threshold (Modified Bonferroni, *p*-value < 0.0002). Attempting to use more stringent thresholds, e.g., based on simpleM (*p*-value < 0.000001) [104], resulted in only a single SNP on OAR 17 (17:22,669,197, genome build Oar_v3.1) crossing genome-wide significance for semen percent post-thaw motility, and none of the other SNPs were found to be associated with any of the other traits at the genome-wide level. This is consistent with the previously published GWAS in sheep, where only one of three semen traits had QTLs that were able to achieve genome-wide significance based on a false discovery rate of 10% [6]. In the same context, a past GWAS focused on semen traits in Chinese Holsteins also used a relatively lenient threshold when the use of a Bonferroni-based threshold did not yield significant QTLs, likely due to high type II errors [105]. The use of a relatively lenient threshold subsequently resulted in the identification of 19 SNPs that were significantly associated with Chinese Holstein bull semen traits, including volume, percent motility, concentration, number of spermatozoa, and number of motile spermatozoa.

In the present study, significant SNPs associated with semen volume, gross motility, concentration, and percent post-thaw motility accounted for 17.08%, 7.59%, 4.69%, and 28.11% of the additive genetic variance (Table 2). Previous GWAS in livestock species have also reported the proportion of genetic variance explained by QTLs found to be significantly associated with semen traits. In cattle, SNPs significantly associated with total motility were found to account for 15.98% of additive genetic variance in crossbred bulls [106], and in another study in dairy cattle, a single SNP significantly associated with semen volume was found to account for 12.22% of the additive genetic variance [107]. In pigs, a GWAS involving almost 100,000 semen collection records from 2022 pigs identified SNPs significantly associated with volume, concentration, and percent motility, accounting for 2.23% and 2.48%, 1.95%, and 6.15% of genetic variance, respectively [108]. Overall, the proportion of genetic variance explained by significant QTLs in any study is likely influenced by a variety of factors, including the genetic background of the resource population and the degree of environmental variance associated with the phenotypes in question. The semen quality data used in this study was collected over a roughly 20 year period and was hence subject to significant environmental variance, which likely contributed to the low heritability associated with these phenotypes. However, this study also involved sheep from multiple environmental locations and management practices, indicating a relatively high degree of genetic variance that likely contributed to individual SNPs explaining a proportion of the additive genetic variance that is largely consistent with previous reports in livestock species. Overall, the QTLs and associated candidate genes identified in the present study could provide insights into the underlying biological mechanisms influencing ram semen traits and possibly be used to develop future strategies to improve the reproductive efficiency of sheep.

It is worth noting that the resource population used in the current study represents a subset of animals that have previously been used to estimate genetic parameters associated with semen traits [22]. The animals included in this study represent the subset of animals for which genotype data was available. Therefore, while heritability of semen traits has been estimated in both studies, they differ in terms of the breed(s) of sheep used, the size of the resource population (n = 11,470 vs. n = 4506), as well as the method used to estimate the relationship between animals in the population (Pedigree vs. GRM). Despite these differences, the heritability estimates computed in both studies are comparable (Table 4) and are also largely in alignment with previous sheep studies [21,22,23,24]. Comparable low to moderate estimates of heritability for semen traits have also been reported in a range of livestock species such as cattle [109,110], goats [111], and pigs [112,113]. Given that the dataset was collected over a roughly 20 year period, environmental variability likely had a significant effect on the variability observed in semen traits. Furthermore, while standardised procedures for assessment of semen traits were used across the collection period, it is likely that subjectivity associated with different technicians that assessed semen during the collection period also contributed to the variability in observed phenotypes. The existence of technician bias, especially for semen traits like percent post-thaw motility, has been previously reported [114]. Overall, this would all contribute to increased environmental variance, which aligns with the low heritability estimates of all semen traits reported in this study. These results indicate that while most of the variability observed in semen traits is environmental in nature, genetic variants do contribute to this variability, and therefore, identifying such genetic variants would afford valuable insights into the physiological drivers underlying these traits, thereby contributing to the development of novel opportunities to improve reproductive efficiency.

## 5. Conclusions

Overall, 35 QTL regions located on 16 OARs were significantly associated with Merino ram semen volume, gross motility, concentration, and percent post-thaw motility. Several genes identified in the present study, including *SORD*, *SH2B1*, and *NT5E*, have been previously found to play crucial roles in spermatogenesis, spermatozoal motility, and high motility following cryopreservation; they may be promising candidate genes for Merino ram semen traits in future studies. Several genes like *PADI2*, *RAB3B*, and *ALDOA*, which have been previously associated with promoting spermatozoal maturation, the acrosome reaction, and conception success, were also identified. As such, it would be beneficial to validate such putative candidate genes via molecular analysis to characterise their influence on semen traits and possibly conception outcomes. Overall, results indicate that Merino semen traits are lowly heritable and that several QTLs influencing variability in these traits exist. Further studies are needed to characterise these QTL regions and the genes contained within them.

## Figures and Tables

**Table 1:** Descriptive statistics of semen traits, including volume, gross motility, concentration, and percent post-thaw motility, provided on rams with genotype records.

	Volume	Gross Motility	Concentration	Percent Post-Thaw Motility
Number of Rams	246	246	245	242
Number of Records	4506	4486	3758	3100
Mean (±Standard Deviation)	1.24 (±0.48)	4.35 (±1.15)	4319.75 (±859.41)	55.25 (±15.34)

Note: Volume of ejaculate in millilitres (mL); gross motility (score from 0 to 5); concentration (spermatozoa × 10^6^); and percent post-thaw motility (%).

**Table 2:** Significant single nucleotide polymorphisms (SNPs) associated with semen traits (volume, gross motility, concentration, and percent post-thaw motility).

Trait	OAR	Location (Mb)	$V_{SNP}\%$	Start Window (Mb)	End Window (Mb)	*p*-Value
Volume	3	8.91	3.14	8.41	9.41	5.70 × 10^5^
	5	35.60	6.90	35.10	36.10	1.34 × 10^4^
	9	53.65	0.72	53.15	54.15	1.11 × 10^4^
	10	44.48	0.07	43.98	44.98	1.92 × 10^4^
	11	25.38	4.61	24.88	25.88	3.68 × 10^5^
	12	48.41	0.47	47.91	48.91	1.09 × 10^5^
	12	50.58	0.65	50.08	51.08	1.75 × 10^5^
	25	7.62	0.48	7.12	8.12	5.19 × 10^5^
	25	30.55	0.04	30.05	31.05	1.74 × 10^5^
Gross Motility	2	195.43	0.41	194.93	195.93	9.07 × 10^5^
	3	6.18	0.21	5.68	6.68	4.64 × 10^5^
	8	50.82	2.30	50.32	51.32	9.03 × 10^5^
	10	16.62	2.03 × 10^3^	16.12	17.12	3.56 × 10^5^
	14	6.22	1.67	5.72	6.72	1.36 × 10^4^
	15	42.21	1.79	41.71	42.71	1.39 × 10^4^
	24	26.44	1.21	25.94	26.94	1.35 × 10^4^
Concentration	2	78.88	0.98	78.38	79.38	1.25 × 10^4^
	2	221.22	0.16	220.72	221.72	8.82 × 10^5^
	2	247.87	0.02	247.37	248.37	1.11 × 10^4^
	3	49.95	0.41	49.45	50.45	1.35 × 10^4^
	5	38.41	0.02	37.91	38.91	1.88 × 10^4^
	7	55.95	1.29	55.45	56.45	1.43 × 10^4^
	20	42.51	1.70	42.01	43.01	1.94 × 10^5^
	25	0.74	0.09	0.24	1.24	1.69 × 10^4^
Percent Post-Thaw Motility	1	25.78	0.22	25.28	26.28	1.32 × 10^4^
	7	49.04	0.84	48.54	49.54	4.31 × 10^5^
	7	62.09	1.67 × 10^4^	61.59	62.59	1.76 × 10^4^
	8	76.70	0.39	76.20	77.20	7.72 × 10^6^
	9	53.42	8.36	52.92	53.92	1.10 × 10^5^
	9	57.73	4.80 × 10^3^	57.23	58.23	1.95 × 10^6^
	9	79.08	0.16	78.58	79.58	1.79 × 10^4^
	14	3.61	13.70	3.12	4.12	8.71 × 10^5^
	15	13.90	0.48	13.40	14.40	1.96 × 10^4^
	17	22.67	1.47	22.17	23.17	3.62 × 10^7^
	25	13.35	2.47	12.85	13.85	1.09 × 10^4^

OAR: *Ovis aries* autosome; Mb: Megabase; V_SNP_%: SNP variance.

**Table 3:** Significant unique candidate genes associated with QTL regions associated with semen volume, gross motility, concentration, and percent post-thaw motility.

Trait	QTL Region (OAR:Mb)	Candidate Gene Symbol
Volume	3:8.41–9.41	*TTC16 ^A^*, *PTRH1*, *C3H9orf117*, *STXBP1 ^A^*, *FAM129B ^A^*, *LRSAM1 ^A^*, *RPL12 ^A^*, *ZNF79*, SLC2A8 *^A^*, *GARNL3*, *RALGPS1 ^A^*, *ANGPTL2 ^A^*, *ZBTB34 ^A^*, *ZBTB43 ^A^*, and *LMX1B ^A^*
	5:35.1–36.1	*COMMD10*, *ARL10*, *NOP16 ^A^*, *HIGD2A ^A^*, *CLTB ^A^*, *FAF2*, *RNF44 ^A^*, *CDHR2 ^A^*, *GPRIN1*, *SNCB*, *EIF4E1B*, *TSPAN17*, *UNC5A*, *HK3 ^A^*, *UIMC1*, *ZNF346*, *FGFR4 ^A^*, and *NSD1 ^A^*
	9:53.15–54.15	-
	10:43.98–44.98	*KLHL1 ^A^*
	11:24.88–25.88	*KIAA0753*, *PITPNM3*, *TRNAK-UUU ^A^*, *FAM64A*, *AIPL1*, *WSCD1*, *TRNAW-CCA ^A^*, *NLRP1 ^A^*, *TRNAC-GCA ^A^*, *MIS12 ^A^*, *DERL2 ^A^*, *DHX33*, *C1QBP ^A^*, *RPAIN ^A^*, and *NUP88 ^A^*, *RABEP1 ^A^*
	12:47.91–48.91	*PRDM16 ^A^*, *ACTRT2 ^A^*, *TRNAC-GCA ^A^*, *TTC34*, *MMEL1 ^A^*, *FAM213B*, *HES5 ^A^*, *PANK4*, *PLCH2 ^A^*, *PEX10 ^A^*, *RER1*, *MORN1*, *SKI*, *C12H1orf86*, *PRKCZ ^A^*, *GABRD ^A^*, *CFAP74*, *TMEM52 ^A^*, and *CALML6*
	12:50.08–51.08	*CASP9 ^A^*, *EFHD2 ^A^*, *FHAD1*, and *TMEM51 ^A^*
	25:7.12–8.12	*SLC35F3 ^A^*, *TARBP1 ^A^*, *IRF2BP2 ^A^*, *TRNAR-CCU*, *TOMM20 ^A^*, *RBM34 ^A^*, *ARID4B ^A^*, and *GGPS1 ^A^*
	25:30.05–31.05	*ADK*, *KAT6B ^A^*, *DUPD1*, *DUSP13 ^A^*, *SAMD8 ^A^*, *VDAC2 ^A^*, *COMTD1*, *ZNF503 ^A^*, and *C25H10orf11*
Gross Motility	2:194.93–195.93	-
	3:5.68–6.68	*FIBCD1 ^A^*, *QRFP ^A^*, *ABL1 ^A^*, *EXOSC2 ^A^*, *PRDM12*, *FUBP3*, *ASS1 ^A^*, *HMCN2*, *NCS1*, *GPR107*, *FNBP1*, *USP20*, *C3H9orf78*, *TOR1A*, and *TOR1B*
	8:50.32–51.32	*SYNCRIP*, *SNX14*, and *NT5E ^A^*
	10:16.12–17.12	*SPERT ^A^*, *SIAH3*, *ZC3H13 ^A^*, *CPB2*, *LCP1 ^A^*, *LRRC63*, *KIAA0226L*, *LRCH1*, *ESD*, and *TRNAG-CCC ^A^*
	14:5.72–6.72	*MAF*
	15:41.71–42.71	*TRNAW-CCA ^A^*, *RNF141 ^A^*, *AMPD3*, *ADM*, *SBF2*, *SWAP70 ^A^*, *WEE1 ^A^*, and *ZNF143*
	24:25.94–26.94	*ATP2A1*, *SH2B1 ^A^*, *TUFM ^A^*, *ATXN2L ^A^*, *CLN3*, *APOBR ^A^*, *IL27 ^A^*, *NUPR1 ^A^*, *CCDC101 ^A^*, *SLX1A*, *BOLA2B*, *CORO1A*, *MAPK3 ^A^*, *GDPD3*, *YPEL3 ^A^*, *TBX6*, *PPP4C ^A^*, *ALDOA ^A^*, *FAM57B*, *C24H16orf92*, *TRNAG-CCC ^A^*, *DOC2A*, *INO80E*, *HIRIP3*, *TAOK2 ^A^*, *TMEM219*, *KCTD13 ^A^*, *ASPHD1*, *SEZ6L2*, *CDIPT ^A^*, *MVP*, *PAGR1*, *PRRT2*, *MAZ*, *KIF22 ^A^*, *ZG16 ^A^*, *C24H16orf54*, *QPRT*, *SPN*, *CD2BP2*, *TBC1D10B ^A^*, *MYLPF*, *ZNF48 ^A^*, *ZNF771 ^A^*, *DCTPP1*, *SEPHS2 ^A^*, *ITGAL ^A^*, *ZNF768*, *ZNF688*, *ZNF689 ^A^*, *PRR14*, *FBRS ^A^*, *SRCAP ^A^*, *TMEM265*, *TRNAR-UCG*, *TRNAR-UCG*, *PHKG2 ^A^*, *C24H16orf93*, *RNF40 ^A^*, and *ZNF629*
Concentration	2:78.38–79.38	-
	2:220.72–221.72	-
	2:247.37–248.37	*ACTL8 ^A^*, *ARHGEF10L ^A^*, *RCC2 ^A^*, *PADI6 ^A^*, *PADI3 ^A^*, *PADI1 ^A^*, and *PADI2 ^A^*
	3:49.45–50.45	-
	5:37.91–38.91	*TRIM52*, *ZNF496 ^A^*, *NLRP3 ^A^*, *GCSAML ^A^*, *TRIM58*
	7:55.45–56.45	*TMOD3 ^A^*, *TMOD2*, *LYSMD2*, *SCG3 ^A^*, *DMXL2 ^A^*, *GLDN*, *CYP19 ^A^*, *TNFAIP8L3 ^A^*, and *AP4E1 ^A^*
	20:42.01–43.01	*SIRT5*, *GFOD1*, *TBC1D7*, and *PHACTR1 ^A^*
	25:0.24–1.24	*RAB4A ^A^*, *SPHAR*, *CCSAP ^A^*, *ACTA1 ^A^*, *NUP133 ^A^*, *ABCB10 ^A^*, *TAF5L ^A^*, and *URB2 ^A^*
Percent Post-Thaw Motility	1:25.28–26.28	*C1H1orf185*, *RNF11 ^A^*, *TRNAY-GUA*, *TTC39A*, *EPS15 ^A^*, *OSBPL9 ^A^*, *NRD1 ^A^*, *RAB3B ^A^*, *TXNDC12 ^A^*, *KTI12*, and *BTF3L4 ^A^*
	7:48.54–49.54	*ADAM10*, *LIPC*, *AQP9 ^A^*, and *ALDH1A2 ^A^*
	7:61.59–62.59	*SQRDL ^A^*, *BLOC1S6 ^A^*, *SLC30A4 ^A^*, *C7H15orf48*, *SPATA5L1*, *GATM ^A^*, *SLC28A2 ^A^*, *TRNAH-GUG*, *SHF ^A^*, *DUOX1 ^A^*, *DUOXA1 ^A^*, *DUOXA2 ^A^*, *DUOX2 ^A^*, *SORD ^A^*, and *FERMT2 ^A^*
	8:76.2–77.2	*SYNE1 ^A^*, *MYCT1 ^A^*, *VIP ^A^*, *FBXO5 ^A^*, *MTRF1L ^A^*, and *RGS17 ^A^*
	9:52.92–53.92	*PEX2 ^A^*
	9:57.23–58.23	*FABP5 ^A^*, *PMP2 ^A^*, *FABP9 ^A^*, *FABP4 ^A^*, *FABP12 ^A^*, *NOV*, *MAL2*, *COLEC10*, and *TNFRSF11B ^A^*
	9:78.58–79.58	*NIPAL2*, *POP1*, *HRSP12 ^A^*, *ERICH5*, *RPL30 ^A^*, *MATN2 ^A^*, *LAPTM4B ^A^*, *MTDH ^A^*, and *TSPYL5 ^A^*
	14:3.12–4.12	*MON1B ^A^*, *SYCE1L ^A^*, and *ADAMTS18 ^A^*
	15:13.4–14.4	*MAML2*, *MTMR2 ^A^*, *CEP57 ^A^*, and *FAM76B ^A^*
	17:22.17–23.17	-
	25:12.85–13.85	*ZNF25 ^A^*, *ZNF33B ^A^*, *ZNF248*, *BMS1*, *TRNAE-UUC*, *RET*, *CSGALNACT2 ^A^*, *RASGEF1A ^A^*, *FXYD4 ^A^*, *HNRNPF ^A^*, and *BICC1*

The genes with a superscript ^A^ were previously found to be associated with spermatogenesis and male fertility; OAR: *Ovis aries* autosome; Mb: Megabase.

**Table 4:** Variance components and heritability estimates for semen traits including volume, gross motility, concentration, and percent post-thaw motility.

Trait	$\sigma_{a^{2}}$	$\sigma_{c^{2}}$	$\sigma_{d^{2}}$	$\sigma_{e^{2}}$	$h^{2}$	r (± SE)
Volume	0.005	0.006	0.010	0.027	0.104	0.436 (±0.170)
Gross Motility	0.011	0.013	0.011	0.044	0.139	0.443 (±0.161)
Concentration	3.610	4.230	10.981	31.692	0.071	0.373 (±0.186)
Percent Post-Thaw Motility	0.106	0.109	0.224	0.707	0.092	0.383 (±0.182)

Note: $\sigma_{a^{2}}$: additive genetic variance; $\sigma_{c^{2}}$: permanent environmental variance; $\sigma_{d^{2}}$: stud-year variance; $\sigma_{e^{2}}:$ residual variance; $h^{2}$: heritability; r: repeatability; and SE: standard error.

## Data Availability

The phenotype data present in this study is available upon request from the corresponding author, while the genotype data is confidential and covered under a data sharing agreement with Charles Sturt University (CSU) and Meat and Livestock Australia (MLA).

## Supplementary Materials

The following supporting information can be downloaded at: https://www.mdpi.com/article/10.3390/ani13142286/s1, Table S1: Significance of fitted effects for each semen volume, gross motility, concentration, and percent post-thaw motility; Table S2: Uncharacterised genes associated with each QTL identified in semen volume, gross motility, concentration, and percent post-thaw motility.
