# Supplementary material for: QTLs and Candidate Genes Associated with Semen Traits in Merino Sheep
Source: Animals (Basel). 2023 Jul 12;13(14):2286. doi: 10.3390/ani13142286 (PMC10376747; doi:10.3390/ani13142286)
Supplement: Supplementary file 1 [file animals-13-02286-s001.zip › animals-2459823-supplementary.pdf]

**Supplementary Table S1**: Significance of fitted effects for each semen volume, gross motility, concentration, and percent post-thaw motility

| | Volume | Gross Motility | Concentration | Percent Post-Thaw Motility |
|---|---|---|---|---|
| Age at collection | *** | *** | *** | *** |
| Collection centre | *** | *** | *** | *** |
| Collection number | *** | *** | NS | NS |
| Breed season | *** | * | *** | ** |
| Collection method | NS | ** | NS | *** |

Note: *** = ($p < 0.001$), ** = ($p < 0.01$), * = ($p < 0.05$), and NS = ($p > 0.05$)

**Supplementary Table S2**: Uncharacterised genes associated with in each QTL identified in semen volume, gross motility, concentration, and percent post-thaw motility.

| **Trait** | **QTL Region (OAR:Mb)** | **Candidate Gene Symbol** |
|---|---|---|
| **Volume** | 3:8.41-9.41 | *LOC105612588*, *LOC105612590*, *LOC105612594*, *LOC105612621* |
| | 5:35.1-36.1 | *LOC105615251*, *LOC101116567*, *LOC105615252*, *LOC105613755*, *LOC105613754*, *LOC105611543*, *LOC105611545*, *LOC105611544*, *LOC101119981*, *LOC105611546*, *LOC105611547* |
| | 9:53.15-54.15 | *LOC105613955* |
| | 10:43.98-44.98 | *LOC101121784* |
| | 11:24.88-25.88 | *LOC105610742*, *LOC105616326*, *LOC105616457*, *LOC105616327* |
| | 12:47.91-48.91 | *LOC105616533*, *LOC101103167*, *LOC105609714*, *LOC105609715*, *LOC105609719*, *LOC105616534*, *LOC101121791*, *LOC105616535*, *LOC101123057* |
| | 12:50.08-51.08 | *LOC101111210*, LOC105616539, *LOC105616540*, *LOC101110432*, *LOC105616541*, *LOC105616542*, *LOC105616543*, *LOC105610224*, *LOC105616544* |
| | 25:7.12-8.12 | *LOC105604913*, *LOC101113195*, *LOC105604914*, *LOC101113650*, *LOC105604915*, *LOC105604917*, *LOC105604916*, *LOC105604918* |
| | 25:30.05-31.05 | *LOC105604997*, *LOC105605080*, *LOC105605000*, *LOC105605091*, *LOC105605001*, *LOC105605002*, *LOC105605003* |
| **Gross Motility** | 2:194.93-195.93 | *LOC101122844* |
| | 3:5.68-6.68 | *LOC105607238*, *LOC105612447*, *LOC105607239*, *LOC105607240* |
| | 8:50.32-51.32 | *LOC105609780*, *LOC105611302*, *LOC105611301*, *LOC105615880*, *LOC105611299*, *LOC105611298* |
| | 10:16.12-17.12 | *LOC105616140*, *LOC105608006*, *LOC105616141*, *LOC105616142*, *LOC105616143* |

| | | |
|---|---|---|
| | 14:5.72-6.72 | *LOC105616821, LOC105616820, LOC105609368, LOC105616822, LOC105609367, LOC105609365, LOC105616823, LOC105609364, LOC105609363* |
| | 15:41.71-42.71 | *LOC105602253, LOC105602254, LOC105602255, LOC101104267* |
| | 24:25.94-26.94 | *LOC105604726, LOC105604725, LOC101112443, LOC101112694, LOC105604727, LOC101114075, LOC105604728, LOC105604730, LOC105604731, LOC105604732, LOC101102954, LOC101103208, LOC105604733, LOC105604736, LOC105604735, LOC101122752, LOC105604737* |
| **Concentration** | 2:78.38-79.38 | *LOC105608114* |
| | 2:220.72-221.72 | *LOC105606755, LOC101118589, LOC101115609, LOC105606754, LOC105606772* |
| | 2:247.37-248.37 | *LOC105611563, LOC105612023, LOC105611566, LOC105611567, LOC105610920, LOC101107939, LOC105610921, LOC105610922* |
| | 3:49.45-50.45 | *LOC101120736, LOC101120996, LOC654331, LOC101122016, LOC101121246* |
| | 5:37.91-38.91 | *LOC101112480, LOC105614551, LOC101112729, LOC105615341, LOC105613511, LOC101113495, LOC105613512, LOC101107959, LOC101108225, LOC101113757, LOC101108485, LOC101114017, LOC101114270, LOC101108746, LOC101114521, LOC101109012, LOC101114777, LOC101115031, LOC101115277, LOC101109269, LOC101109535, LOC101115539, LOC101115794, LOC101116054, LOC101116574, LOC101116831, LOC101117077, LOC101117332, LOC101117585* |
| | 7:55.45-56.45 | *LOC105607297, LOC105615718, LOC105607294, LOC105607293* |
| | 20:42.01-43.01 | *LOC105603856, LOC105603857, LOC105603859, LOC105603858, LOC105603860, LOC105603861, LOC105603863, LOC105603862* |
| | 25:0.24-1.24 | *LOC101123180, LOC105604888, LOC105604889, LOC105604890* |
| **Percent Post-Thaw Motility** | 1:25.28-26.28 | *LOC105609503, LOC105605441, LOC105609505, LOC105609934, LOC105609504, LOC105609507, LOC105609506, LOC105610201, LOC105609513, LOC105609508* |
| | 7:48.54-49.54 | *LOC105609699, LOC105615709, LOC105609702* |
| | 7:61.59-62.59 | *LOC101113241, LOC105615725, LOC105615726, LOC105612095, LOC105615727, LOC105612097, LOC105612096, LOC101112487, LOC101115542, LOC105612098* |
| | 8:76.2-77.2 | *LOC105615930, LOC101106779, LOC101104501* |
| | 9:52.92-53.92 | *LOC105611155, LOC105611156, LOC105613955* |
| | 9:57.23-58.23 | *LOC105616058, LOC105611122, LOC105613108, LOC105611121, LOC105616059, LOC105613105* |
| | 9:78.58-79.58 | *LOC105616083, LOC105616084, LOC105611867, LOC105611866, LOC101107532, LOC105611865* |

| | | |
|---|---|---|
| | 14:3.12-4.12 | *LOC105613905*, *LOC105602052*, *LOC105609376*, *LOC105616809*, *LOC105616810*, *LOC105616811*, *LOC105616812* |
| | 15:13.4-14.4 | *LOC105602135*, *LOC105602134*, *LOC105602133*, *LOC105602132*, *LOC105602131*, *LOC105602137* |
| | 17:22.17-23.17 | - |
| | 25:12.85-13.85 | *LOC105604934*, *LOC105604933*, *LOC105604932*, *LOC105601946*, *LOC101118756*, *LOC105604929*, *LOC101119276*, *LOC105605087*, *LOC101119530*, *LOC105604935*, *LOC105604936* |

Note: OAR: *Ovis aries* autosome; Mb: Mega base
